# Vet the journal before you submit: turnaround times of journals publishing in zoological medicine and related fields

**DOI:** 10.7717/peerj.15656

**Published:** 2023-07-11

**Authors:** Brendan Runde, Craig Harms

**Affiliations:** 1 Department of Applied Ecology, College of Agriculture and Life Sciences, Center for Marine Sciences and Technology, North Carolina State University, Morehead City, NC, United States of America; 2 The Nature Conservancy, Charlottesville, VA, United States of America; 3 Department of Clinical Sciences, College of Veterinary Medicine, Center for Marine Sciences and Technology, North Carolina State University, Morehead City, NC, United States of America

**Keywords:** Journal selection factors, Time of first decision, Time to acceptance, Time to publication, Transparency in review times, Turnaround time

## Abstract

Many factors influence selection of a target journal for publishing scientific papers, including “fit” within the journal’s scope, acceptance rate, readership, open access options, submission and publication costs, journal quality, and timeliness of publication. Timeliness of publication can be a critical factor affecting career development, but many journals are not transparent about turnaround times. Here we evaluated 49 journals publishing papers in zoological medicine and related fields between 2017 and 2022, and aggregated and examined distributions of turnaround time of journals that publicly provided the requisite data, in order to aid authors in selecting target journals that best meet their needs. Of 49 journals evaluated, 39 provided necessary dates for reconstructing turnaround times. Of these, median times to acceptance ranged from 37 to 338 days, and median times to publication ranged from 41 to 403.5 days. The percentage of papers published in greater than 1 year (“slow”) ranged from 0 to 57.1%, while the percentage of papers published in under 6 months (“timely”) ranged from 0.8 to 99.8%. Acceptance rates and times to first decision were available for only 22% and 20%, respectively, of journals evaluated. Results may prove useful for authors deciding where to submit their works, depending on how they prioritize the many factors involved.

## Introduction

Selecting a target journal for a scientific manuscript can be a difficult decision. Important factors include the paper’s “fit” within the journal’s scope, likelihood of acceptance, target readership, open access options, submission and publication costs, a measure of journal quality such as impact factor, less easily quantified qualities such as review quality and editorial management, and timeliness of publication. Unfortunately, many journals and publishers are less-than-transparent about some of these factors—such as turnaround time and acceptance rate—leaving authors to rely on anecdotal and limited experiential information.

Choosing a journal that does not meet one’s needs and expectations can be professionally detrimental, as producing peer-reviewed research within set time frames is important to career advancement in many fields. For instance, in zoological medicine (construed broadly to include zoo, wildlife, aquatic, and exotic animal medicine) and other disciplines, publishing is important for students and early career professionals to compete successfully for internships and residencies, credentialing for specialty boards, job applications, and promotion and tenure decisions. Training programs often set expectations for number of publications within the time frame of the internship or residency as a condition of awarding certificates of completion, and academic institutions have expectations for scholarly productivity within a defined timeline in order to be considered for promotion and tenure, which can be particularly challenging for faculty with clinical service responsibilities to meet (San Miguel, 2019). Because early career research results often naturally manifest towards latter stages of student years, internship and residency programs, and pre-tenure time lines, prolonged turnaround time for a paper may cause delays in professional benchmarks, such as specialty board credentialing and tenure. Time to acceptance is a critical factor for individuals compiling credentials packets for the American College of Zoological Medicine (ACZM) examination, as credentialling requires that the applicant “be first author on at least three (3) publications relevant to the field of zoological medicine in refereed journals,” and “the manuscript must be fully accepted for publication prior to the deadline for applying for the certification examination” (American College of Zoological Medicine, 2022). Similar conditions apply for individuals pursuing board certification in the European College of Zoological Medicine—Zoo Health Management (ECZM (ZHM)), for which the applicant must be author of “three (3) original peer-reviewed scientific papers in a well-established internationally refereed scientific journal…,” and “must be the principal author” of at least two of those (European College of Zoological Medicine, 2020). These conditions may place a premium on expeditious manuscript review at any career stage for an individual pursuing ACZM or ECZM (ZHM) board certification, but particularly so for third-year residents in zoological medicine programs aiming to sit one of these board examinations immediately following their residency.

Inaccessible data on manuscript turnaround times hinder informed journal submission decisions by authors in time-sensitive situations. Some journals that publish papers in zoological medicine and related fields provide per-paper publication histories (*i.e.,* date received, date accepted, and date published) that allow for the determination of individual turnaround times. Aggregating turnaround times for many papers would allow for the generation of per-journal statistics that could be used by authors to refine their decision of which journal to target. Turnaround time statistics for over 80 journals that publish papers in fisheries science were recently provided (Runde, 2021). Here, we evaluate 49 journals that publish papers in zoological medicine and related fields, including those on the ACZM Annotated Suggested Reading List (American College of Zoological Medicine, 2022) and the ECZM (ZHM) reading list (European College of Zoological Medicine, 2020), with the same goal as the fisheries science paper (Runde, 2021): to aid authors in selecting target journals that best meet their needs and expectations.

## Materials & Methods

We developed a list of journals for inclusion in this study based on all journals on the ACZM and ECZM (ZHM) reading lists from 2021 and 2022; these lists were supplemented with related journals in the field based on the authors’ knowledge (in particular, CAH, diplomate of ACZM and ECZM (ZHM), Past President of ACZM, 30 years in the field). The final list was comprised of 49 journals, including some that do not publish exclusively in the field of zoological medicine (*e.g., Journal of the American Veterinary Medical Association*) or even of veterinary medicine (*e.g*., *PeerJ*; Table 1), but which nevertheless publish relevant papers and in several instances are included on ACZM and ECZM (ZHM) reading lists. Some higher impact journals that occasionally contain papers of zoological medicine and related field interest (*e.g*., *Science*, *Nature*) are not included here because of their broader scope and their inclusion in a prior paper (Runde, 2021), while some journals included in that study more relevant to the current paper are included with updated data.

**Table 1:** Summary data for full suite of 49 journals examined.

Journal	IF	2021 ACZM	2022 ACZM	2022 ECZM ZHM	Turnaround time obtained?	Acceptance rate	Days to first decision
American Journal of Veterinary Research	0.9	Core	Core	X	Yes		
Animals	3.1				Yes		
BMC Veterinary Research	2.7				Yes		
Canadian Veterinary Journal	0.6				Not Reported		
Chelonian Conservation and Biology	1.1				Yes		
Conservation Biology	6.7	Additional			Yes	0.15	55
Conservation Physiology	2.8				Yes		
Diseases of Aquatic Organisms	1.7	Additional	Additional		Yes		
Emerging Infectious Diseases	9.9	Additional	Additional		Not Reported		
Fish and Shellfish Immunology	4.3				Yes	0.44	35
Frontiers in Veterinary Science	3.2				Yes		
Herpetological Conservation and Biology	1.1				Yes		
Journal of Aquatic Animal Health	2.5	Additional	Additional		Yes		
Journal of Avian Medicine and Surgery	0.4	Core	Core	X	Not Reported		
Journal of Exotic Pet Medicine	0.5	Additional	Additional		Not Reported	0.41	21
Journal of Fish & Wildlife Management	0.8				Yes		
Journal of Fish Diseases	2.6				Yes		
Journal of Herpetological Medicine and Surgery	NL	Core	Core	X	Not Reported		
Journal of Invertebrate Pathology	2.8				Yes	0.37	42
Journal of Medical Primatology	0.7				Yes		
Journal of Small Animal Practice	1.4	Additional	Additional		Yes		
Journal of the American Association for Laboratory Animal Science	1.4	Additional	Additional		Yes	0.6	
Journal of the American Veterinary Medical Association	0.7	Core	Core	X	Not Reported		
Journal of Veterinary Diagnostic Investigation	1.4				Not Reported		
Journal of Veterinary Medical Education	1.0				Not Reported		
Journal of Veterinary Pharmacology and Therapeutics	1.5				Yes		
Journal of Wildlife Diseases	1.6	Core	Core	X	Yes		
Journal of Wildlife Management	2.3	Additional	Additional		Yes		
Journal of Zoo and Aquarium Research	NL		Additional	X	Yes		
Journal of Zoo and Wildlife Medicine	0.8	Core	Core	X	+/-, no ”dates received”		68
Marine Mammal Science	2.1				Yes		
PeerJ	3.0				Yes	0.42	30
PlosOne	3.6		Additional		Yes	0.47	43
Research in Veterinary Science	2.5				Yes		
The Journal of Veterinary Medical Science	1.1				Yes		
The Veterinary Journal	2.6				Yes		
Theriogenology	2.9				Yes	0.33	38
Veterinary Anaesthesia and Analgesia	1.4				Yes		
Veterinary Clinical Pathology	0.8				Yes		
Veterinary Medicine International	1.7				Yes	0.19	
Veterinary Microbiology	3.2				Yes	0.21	34
Veterinary Ophthalmology	1.5				Yes		
Veterinary Parasitology	2.9				Yes		
Veterinary Pathology	2.5				Not Reported		
Veterinary Quarterly	7.4				Yes	0.25	24
Veterinary Radiology and Ultrasound	1.1				Yes		
Veterinary Record	0.4	Additional	Additional		Yes		
Veterinary Record Open	1.6				Yes		
Zoo Biology	1.6	Additional	Additional	X	Yes		

**Notes.**

Full suite of 49 journals examined, including impact factor (IF), designation as Core or Additional in the suggested reading list for the American College of Zoological Medicine (ACZM) for both 2021 and 2022, inclusion in the suggested reading list of the European College of Zoological Medicine –Zoo Health Management (ECZM ZHM) for 2022, whether or not turnaround time was obtained, acceptance rate (if available), and days to first decision (if available). NL, not listed.

For each journal, we obtained the 2020 impact factor, the most recent year for which it was available for all journals evaluated (Resurchify, 2020). Impact factor is calculated as the number of citations received in a given year by all papers published in that journal during the previous 2 years, divided by the number of papers published in that journal in that timespan. Due to its susceptibility to manipulation, impact factor is considered an imperfect metric of journal quality (Ioannidis & Thombs, 2019; Seglen, 1997), but is still widely relied on by many authors (Archambault & Larivière, 2009; Smith, 2006).

For each journal, we accessed the webpage and/or PDFs of recently published papers and located publication history information (*i.e*., the dates the paper was received, accepted, and published) if it was available. Dates were tabulated for each paper, generally back to the beginning of 2018. Where possible, we excluded publications that were not original research (*e.g*., reviews, brief communications, editorials, errata), on the assumption that such documents have inherently different turnaround times.

We examined distributions of time-to-acceptance (*date accepted*–*date received)* and time-to-publication (*date published*–*date received*) for each journal where information was available. Some papers list multiple publication dates (*i.e., date published online* and *date published in issue*)—we always used whichever publication date came first (generally, *date published online*).

Some papers reported inconceivably short time-to-acceptance or time-to-publication (*e.g.*, received to accepted in 0 d). It is extremely unlikely that a peer-reviewed paper could legitimately be submitted and accepted on the same day. In fact, the authors consider that any acceptance or publication occurring in under 14 d is likely not reflective of the paper’s true timeline. To that end, we eliminated from further analysis any papers accepted or published in 14 d or less from the date received (although we report the proportion excluded on that basis for each journal).

We generated summary data in R 4.1.1 (R Core Team, 2021) for each journal with publication history. Specifically, we focused on median time-to-acceptance and median time-to-publication for each journal. We also evaluated each journal for the proportion of papers published in under 6 months (considered “timely”) and the proportion of papers published in over 12 months (considered “slow”), though we suggest that these metrics be used as general guidance as these cutoffs are arbitrary.

## Results

Of 49 journals included, 39 reported *date received, date accepted,* and *date published* on at least a portion of their published papers (Table 1). One journal (*Journal of Zoo and Wildlife Medicine*) reported *date accepted* and *date published* but not *date received.* The remaining nine journals generally reported only *date published*. One of these (*Journal of the American Veterinary Medical Association*) claims an average time from submission to publication of less than 100 d (Fourtier, 2022), but does not report *date received* or *date accepted*. From these 39 journals, we collected information on 29,054 individual papers.

**Table 2:** Publication histories for the 39 journals that provided requisite information.

Journal	% Under 14 d	Number Of articles	Start date	End date	Median days to acceptance	Median days to publish	% Over 1 year	% Under 6 months
American Journal of Veterinary Research	0.4	277	1/1/2018	9/1/2021	108	337	36.1	1.4
Animals	0.5	4,605	9/28/2020	6/20/2022	37	41	0.0	99.8
BMC Veterinary Research	0	1,369	1/2/2018	6/27/2022	184	202	11.0	41.6
Chelonian Conservation and Biology	0	209	1/1/2015	12/3/2021	140	337	44.5	6.7
Conservation Biology	0	361	3/31/2017	5/16/2021	199	234	14.4	32.1
Conservation Physiology	0.2	618	1/5/2016	7/7/2022	144	182.5	7.4	47.6
Diseases of Aquatic Organisms	2.8	436	3/22/2018	6/23/2022	146	218	7.6	30.3
Fish and Shellfish Immunology	0	2,128	7/27/2017	7/3/2021	91	95	0.4	92.6
Frontiers in Veterinary Science	0	2,007	4/9/2020	6/10/2022	71	110	0.8	86.2
Herpetological Conservation and Biology	0	260	4/30/2017	12/16/2021	236.5	324	41.9	9.2
Journal of Aquatic Animal Health	0	74	12/18/2017	10/11/2021	185.5	253.5	18.9	25.7
Journal of Fish and Wildlife Management	0	197	2/1/2016	4/14/2022	211	212	13.7	28.4
Journal of Fish Diseases	0	480	7/5/2017	2/12/2021	62	105	0.0	93.8
Journal of Invertebrate Pathology	0.2	467	10/24/2017	6/27/2022	128	132	3.2	67.9
Journal of Medical Primatology	0	101	7/24/2018	6/5/2022	113	149	5.0	63.4
Journal of Small Animal Practice	0	415	7/18/2017	8/31/2021	186	254	20.7	24.8
Journal of the American Association for Laboratory Animal Science	0	278	3/1/2018	5/1/2022	101	280.5	10.8	5.4
Journal of Veterinary Pharmacology and Therapeutics	0	305	4/27/2017	5/19/2022	120	154	1.0	63.6
Journal of Wildlife Diseases	0	374	1/1/2018	3/11/2022	133	307	28.9	11.0
Journal of Wildlife Management	0.9	530	11/5/2016	9/21/2021	206	261	22.6	18.3
Journal of Zoo and Aquarium Research	0	68	1/31/2021	7/31/2022	338	403.5	55.9	11.8
Marine Mammal Science	0	274	8/21/2017	3/18/2022	289.5	340.5	41.2	7.7
PeerJ	0	2,681	2/12/2013	7/18/2022	124	158	5.3	61.8
PlosOne	0	2,883	5/16/2018	5/11/2022	164	194	9.2	43.7
Research in Veterinary Science	0.1	915	5/23/2017	7/7/2022	160	167	6.9	54.9
The Journal of Veterinary Medical Science	0	1,297	11/6/2017	3/14/2022	103	120	3.5	75.1
The Veterinary Journal	0	7	3/2/2022	5/11/2022	290	299	28.6	28.6
Theriogenology	3.1	1,938	6/28/2017	7/5/2022	136	141	1.4	70.7
Veterinary Anaesthesia and Analgesia	0	368	4/17/2017	4/29/2022	182	226.5	16.0	32.9
Veterinary Clinical Pathology	0	119	8/17/2018	3/30/2022	151	382	57.1	0.8
Veterinary Medicine International	0	96	11/27/2020	7/1/2022	117.5	137	5.2	63.5
Veterinary Microbiology	0.3	1,134	11/6/2017	8/5/2022	93	98	1.5	87.4
Veterinary Ophthalmology	0	75	2/4/2019	5/5/2022	166	196	9.3	42.7
Veterinary Parasitology	0.3	714	11/2/2017	8/4/2022	109.5	116	1.5	79.8
Veterinary Quarterly	1.6	61	12/1/2014	8/8/2022	176	206	9.8	39.3
Veterinary Radiology and Ultrasound	0.3	384	8/30/2017	6/9/2022	147	224	9.6	23.2
Veterinary Record	0	198	1/6/2018	6/14/2022	181.5	251..5	20.2	10.6
Veterinary Record Open	0	155	1/5/2016	8/2/2022	150	195	16.1	40.6
Zoo Biology	0.5	196	12/6/2017	3/7/2022	233	259	20.9	29.1

**Notes.**

For the 39 journals that provided requisite publication histories: percentage of papers accepted in under 14 days, number of articles analyzed, start and end dates of analyses, median number of days to acceptance and to publication, percentage of papers published in over 1 year from submission (considered prolonged), and percentage of papers published in under 6 months (considered timely).

**Figure 1:**
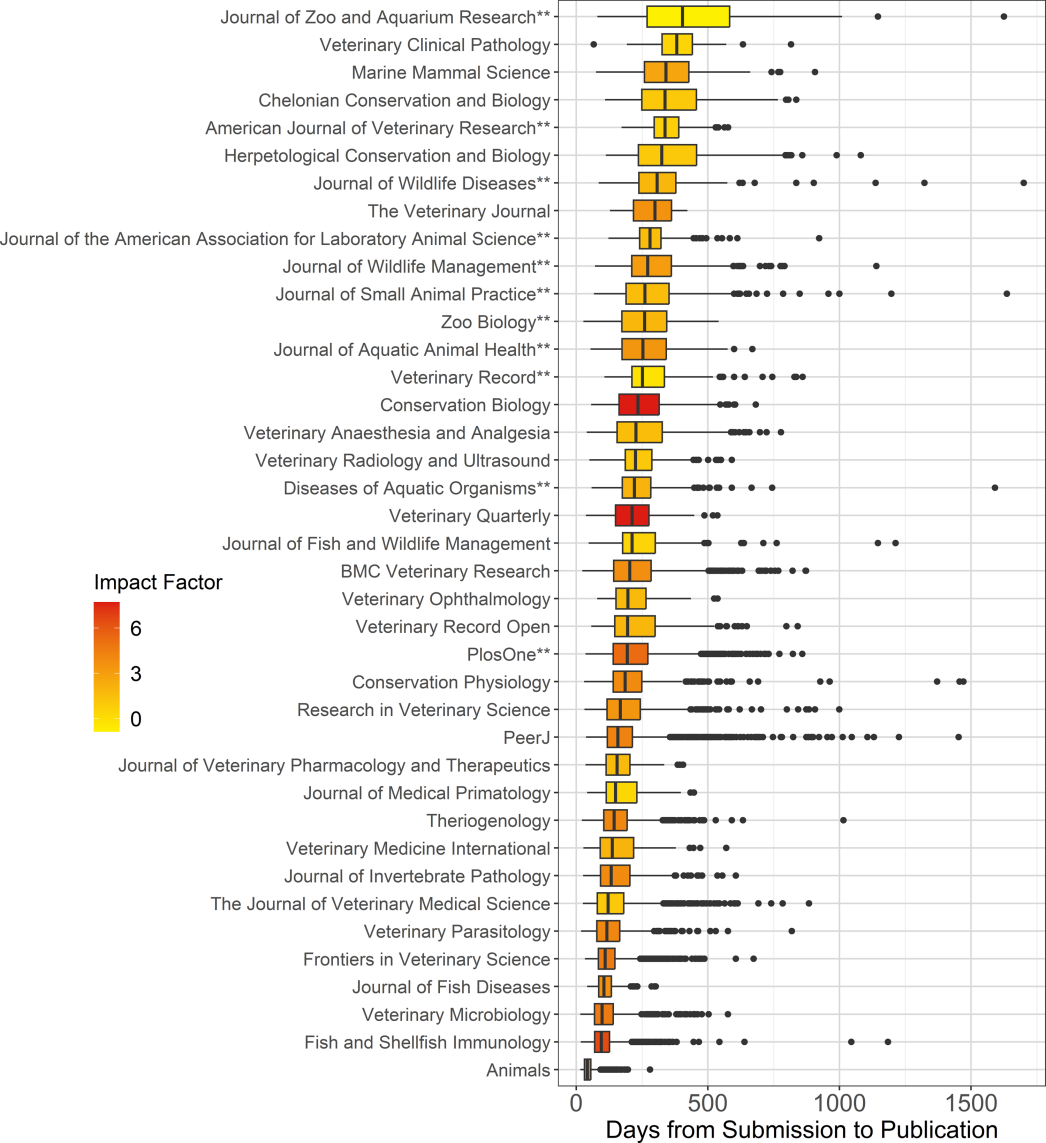
Box plots showing days from submission to publication for 39 journals that publish papers in zoological medicine and related topics organized in descending order of medians. Central vertical lines represent medians, hinges represent the 25th and 75th percentiles, and lower and upper whiskers extend to either the lowest and highest values respectively, or 1.5 * the inter-quartile range. Black dots represent papers that were outside the 1.5 * inter-quartile range. Boxes are shaded to correspond with 2020 Impact Factor, where darker green represents higher impact. Two asterisks (**) = journals included on either 2022 suggested reading lists of the ACZM or ECZM. Journals on ACZM or ECZM reading lists not included in this figure do not provide requisite data on *date received, date accepted,* and *date published*.

For the 39 journals that provided requisite publication histories, median times to acceptance ranged from 37 to 338 d and median times to publication ranged from 41 to 403.5 d (Table 2, Fig. 1). Most of these journals published zero papers in under 14 d from receipt, although 13 journals published between 0.1% and 3.1% of papers in this extremely rapid timeframe (these papers were not included in analyses; see Methods). The percentage of papers for which publication took over 1 year ranged from 0 to 57.1%, while the percentage of papers published in under 6 months ranged from 0.8 to 99.8%. Most journals published at least a few papers that had extremely lengthy turnaround times, ranging up to 1,700 d (Fig. 1).

Acceptance rates and times to first decision were obtained from journal websites or a prior publication^3^ for 11 and 10 journals, respectively. Reported acceptance rates ranged from 15% to 60% and reported times to first decision ranged from 21 d to 68 d (Table 1). We did not conduct further analyses with these data given their scarcity among journals examined.

## Discussion

Over 20% of journals examined do not report one or more dates necessary for reconstructing turnaround times. In the interest of transparency in the scientific publication process, we encourage the publishers and editorial staff of these journals (detailed in Table 1) to report this information. Some progress in this direction may already be occurring since the time frame over which data were collected for this study. Similarly, we encourage more journals to be transparent about acceptance rates and times to first decision. Only 22% of journals considered here report acceptance rates and only 20% report times to first decision.

Journals in the ACZM and ECZM (ZHM) reading lists are selected by diplomates of the respective Colleges’ examination committees for their relevance to zoological medicine as determined through formal job task analyses and examination validation exercises. For journals on the reading lists that are not exclusively focused on zoological medicine (*e.g., American Journal of Veterinary Research, Emerging Infectious Diseases*, *etc*.), only content relevant to zoological medicine is considered as potential examination material. Here we assume that turnaround times do not substantially differ among papers of different topics in these journals. The designation of journals as primary source material for certification examinations could be expected to put a premium on publishing therein, both by diplomates providing content to build the body of zoological medicine literature for dissemination of knowledge to candidates and for question generation, and by candidates who might then have the happy chance of seeing an examination question sourced from their research. Such journals could also reasonably be expected to have a team of the most knowledgeable and dedicated associate editors and reviewers in the field, who can provide the most informed peer review to improve the final manuscript. Similar considerations would apply to other veterinary specialty colleges with publication requirements for credentialing, albeit a lesser number of publications required than ACZM.

Other factors frequently take priority when selecting target journals, however. Interests in some of these factors may converge between residents and faculty mentors, and some may diverge. For instance, publication in a higher impact journal may be prioritized by a junior faculty member with a comfortable timeline to promotion and tenure, while her 3rd year resident under pressure to credential for the board examination that year would prioritize time to acceptance and less restrictive acceptance rates. Median impact factor of the core journals of the combined ACZM and ECZM (ZHM) reading lists is only 0.7, with a range from 0 (or no impact factor designated) to 1.5. Neither ACZM nor ECZM (ZHM) take journal impact factor into account in candidate credentialing, and require only that candidates’ manuscripts “be published in a refereed journal” (American College of Zoological Medicine, 2022) “or” in a well-established internationally refereed scientific journal” (European College of Zoological Medicine, 2020). Both organizations elaborate further on what those criteria entail, with inclusion on the suggested reading lists being a favorable factor. For academic promotion and tenure committee deliberations, however, journal impact factor often does play an explicit role (McKiernan et al., 2019). Note again, however, that impact factor is an imperfect indicator of journal quality (Ioannidis & Thombs, 2019; Runde, 2021; Seglen, 1997). Longer publication intervals common in zoological medicine journals (*e.g.,* quarterly *versus* monthly, weekly or on a continuous rolling basis) can adversely affect both turnaround times and impact factors. Open access can be a common desire, but high fees for open access can be a limiting factor by cutting into grant or personal funds, and have been implicated in leading to inequitable representation of authors from low-income countries (Smith et al., 2022; Solomon & Björk, 2012).

Time to first decision may be the most relevant metric of journal editorial and reviewer efficiency, because it is less affected by author responsiveness to reviews. This metric, however, is even less frequently reported than times to acceptance and publication (just 20% in the journals evaluated here). Further, it does not account for quality of reviews. Review quality and unusually rapid turnaround times are concerns that have been raised particularly with respect to mega-journals and special issues with guest editors, where there may be apparent editorial bias and nepotistic behavior (Scanff et al., 2021) and varying levels of editorial competence of guest editors compared with professional editors of traditional journals (Brainard, 2023; Ioannidis, Pezzullo & Boccia, 2023). The Web of Science recently delisted more than 50 journals from its Master Journal List, based on evaluation of 24 measures of quality that include effective peer review (Brainard, 2023). Some of the delisted journals come from the major open-access publishers Hindawi and MDPI (Brainard, 2023), although not any journals evaluated in the present work as yet. Delisted journals are deprived of an impact factor, which can affect both the journal as a less attractive target for paper submissions, and authors who may be relying on the impact factor metric to bolster their promotion and tenure dossiers.

It is important to acknowledge author responsibilities in ensuring a timely turnaround to publication, including following journal formatting guidelines in the initial submission, and promptly responding to reviews. Here we assume that timeliness and tardiness by authors was roughly equivalent among journals examined.

In recent years, some journals have rendered “reject and resubmit” decisions unto reviewed papers that might historically have been tasked with “major revisions.” Early proponents of this alternative noted that potential benefits could include a quicker turnaround time and slightly better chance of publication (Range & Tingstrom, 1992). However, a byproduct of this process is that the resubmitted version would be entered as a new submission with a new starting date. While this practice is not necessarily carried out with intent to artificially depress turnaround time statistics, it introduces bias in studies such as this one. Unfortunately, we cannot account for this bias but encourage journals to use this decision sparingly and never for manipulation.

As noted previously for fisheries journals (Runde, 2021), turnaround times, acceptance rates, and impact factors reported in this study are not fixed and can change over time. Further, readers and editors acting upon these results may cause some change. Aggregated data in the current study should therefore be considered baseline information for the timespan evaluated. Results may prove useful to authors deciding where to submit their works, depending on how they prioritize the factors involved. Despite our focus on turnaround times, in deciding where we should submit the current manuscript we also prioritized open access, target readership and journal “fit” of a publication that commonly carries zoological medicine topics. This is in accord with recommendations that authors consider “fit” as the most important factor in deciding where to submit their manuscripts (Knight & Steinbach, 2008), followed by other factors that best align with their priorities (Runde, 2021; Solomon & Björk, 2012).

## Conclusions

We evaluated 49 journals that publish papers in zoological medicine and related fields, including those on the ACZM Annotated Suggested Reading List and the ECZM (ZHM) Reading List, and analyzed turnaround times for the 39 of those journals publicly providing the requisite data. Additionally, we have aggregated impact factors, and, where publicly available, acceptance rates and times to first decision. Results will aid authors in selecting target journals that best meet their needs and expectations.

##  Supplemental Information

10.7717/peerj.15656/supp-1 Supplemental Information 1 Data All 29,054 individual articles analyzed.

## Additional Information and Declarations

### Data Availability

The following information was supplied regarding data availability: The raw data are available in the Supplementary File.
